# Ten new complete mitochondrial genomes of pulmonates (Mollusca: Gastropoda) and their impact on phylogenetic relationships

**DOI:** 10.1186/1471-2148-11-295

**Published:** 2011-10-10

**Authors:** Tracy R White, Michele M Conrad, Roger Tseng, Shaina Balayan, Rosemary Golding, António Manuel de Frias Martins, Benoît A Dayrat

**Affiliations:** 1 School of Natural Sciences, University of California, 5200 North Lake Road, Merced, CA 95343, USA; 2 Australian Museum, 6 College Street Sydney NSW 2010 Australia; 3 CIBIO-Açores, Center for Biodiversity and Genetic Resources, Department of Biology, University of the Azores, 9501-801 Ponta Delgada, São Miguel, Azores, Portugal

## Abstract

**Background:**

Reconstructing the higher relationships of pulmonate gastropods has been difficult. The use of morphology is problematic due to high homoplasy. Molecular studies have suffered from low taxon sampling. Forty-eight complete mitochondrial genomes are available for gastropods, ten of which are pulmonates. Here are presented the new complete mitochondrial genomes of the ten following species of pulmonates: *Salinator rhamphidia *(Amphiboloidea); *Auriculinella bidentata, Myosotella myosotis, Ovatella vulcani*, and *Pedipes pedipes *(Ellobiidae); *Peronia peronii *(Onchidiidae); *Siphonaria gigas *(Siphonariidae); *Succinea putris *(Stylommatophora); *Trimusculus reticulatus *(Trimusculidae); and *Rhopalocaulis grandidieri *(Veronicellidae). Also, 94 new pulmonate-specific primers across the entire mitochondrial genome are provided, which were designed for amplifying entire mitochondrial genomes through short reactions and closing gaps after shotgun sequencing.

**Results:**

The structural features of the 10 new mitochondrial genomes are provided. All genomes share similar gene orders. Phylogenetic analyses were performed including the 10 new genomes and 17 genomes from Genbank (outgroups, opisthobranchs, and other pulmonates). Bayesian Inference and Maximum Likelihood analyses, based on the concatenated amino-acid sequences of the 13 protein-coding genes, produced the same topology. The pulmonates are paraphyletic and basal to the opisthobranchs that are monophyletic at the tip of the tree. *Siphonaria*, traditionally regarded as a basal pulmonate, is nested within opisthobranchs. *Pyramidella*, traditionally regarded as a basal (non-euthyneuran) heterobranch, is nested within pulmonates. Several hypotheses are rejected, such as the Systellommatophora, Geophila, and Eupulmonata. The Ellobiidae is polyphyletic, but the false limpet *Trimusculus reticulatus *is closely related to some ellobiids.

**Conclusions:**

Despite recent efforts for increasing the taxon sampling in euthyneuran (opisthobranchs and pulmonates) molecular phylogenies, several of the deeper nodes are still uncertain, because of low support values as well as some incongruence between analyses based on complete mitochondrial genomes and those based on individual genes (18S, 28S, 16S, CO1). Additional complete genomes are needed for pulmonates (especially for *Williamia, Otina*, and *Smeagol*), as well as basal heterobranchs closely related to euthyneurans. Increasing the number of markers for gastropod (and more broadly mollusk) phylogenetics also is necessary in order to resolve some of the deeper nodes -although clearly not an easy task. Step by step, however, new relationships are being unveiled, such as the close relationships between the false limpet *Trimusculus *and ellobiids, the nesting of pyramidelloids within pulmonates, and the close relationships of *Siphonaria *to sacoglossan opisthobranchs. The additional genomes presented here show that some species share an identical mitochondrial gene order due to convergence.

## Background

Elucidating the higher phylogenetic relationships of pulmonate gastropods has remained difficult. A morphology-based phylogenetic analysis revealed a high level of homoplasy and resulted in a poorly-resolved tree [1]. Molecular studies have been based on few individual genes, essentially 18S, 28S, 16S, and COI data [2-5], or few complete mitochondrial genomes [6-8].

**Figure 1:**
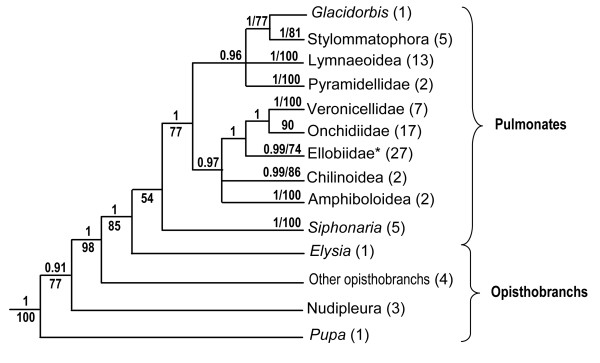
**Phylogenetic relationships of pulmonates**. Relationships obtained in a recent study based on individual genes (18S, 16S, COI), including 79 pulmonate species [5]; the asterisk (*) indicates that the clade Ellobiidae includes three taxa that have not been traditionally regarded as ellobiids (*Otina, Smeagol*, and *Trimusculus*). Node support values are cited using the following format: ''1.00/77'' means that BI posterior probability = 1.00, and that ML bootstrap value = 77%. Only BI posterior probabilities > 0.75 and ML bootstrap values > 50% are shown.

Analyses based on individual gene sequences all provide similar relationships (Figure 1) [2-5]: pulmonates are monophyletic, but they include a few taxa not traditionally classified as pulmonates (Acochlidia, traditionally regarded as opisthobranchs, and Glacidorbidae and Pyramidelloidea, traditionally regarded as basal heterobranchs); also, opisthobranchs are paraphyletic, basal to pulmonates; finally, the false limpet *Siphonaria*, traditionally regarded as a pulmonate, is in some cases found to be more closely related to opisthobranchs than pulmonates. In recent years, taxon sampling has significantly increased in analyses utilizing individual genes: the largest data set (18S, 16S, and COI) established so far includes 79 species representing all major taxa of pulmonates [5].

**Figure 2:**
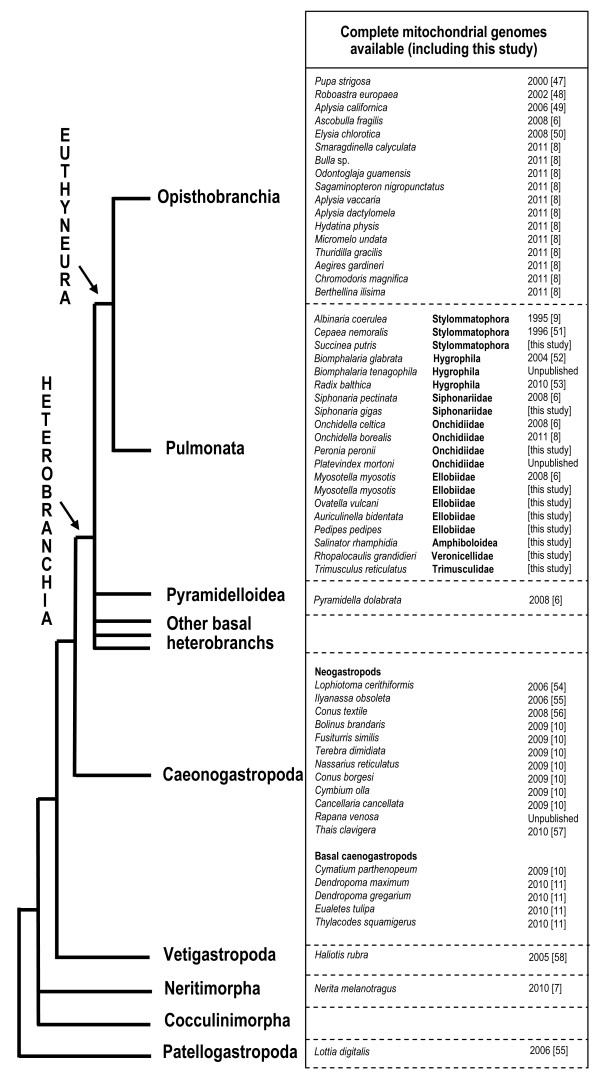
**List of complete gastropod mitochondrial genomes available at present**. The hypothesis of phylogenetic relationships is based on both morphological and molecular data [46]. The reference in which each genome was made available [6-11,47-58] is indicated in brackets as well as the year of publication.

Analyses based on complete mitochondrial genomes provide different phylogenetic relationships, at least for the deep nodes [6-8]: pulmonates are paraphyletic, basal to the monophyletic opisthobranchs; *Siphonaria *is nested within the opisthobranchs. Taxon sampling is still limited in analyses based on complete mitochondrial genomes, mainly because gastropod mitochondrial genomes are still difficult to obtain. Since the first complete gastropod mitochondrial genome was published in 1995 [9], 48 complete genomes have been made available (Figure 2). The use of shotgun sequencing and the decrease in sequencing costs caused a noticeable increase in the production of gastropod mitochondrial genomes a few years ago (Figure 2). Ten genomes became available in nine different publications between 1995 and 2006; since 2008, 38 genomes became available, 30 of which appeared in only four publications [6,8,10,11], although a few papers with only one genome were also published.

At present, Opisthobranchia and Neogastropoda (one of the lineages of Caenogastropoda) are the taxa for which the largest number of complete mitochondrial genomes are available, with 17 and 12 genomes, respectively (Figure 2). Ten genomes are available for pulmonates (including two unpublished), but only some of the pulmonate higher clades are represented. The taxon sampling for the other gastropods is either insufficient (Patellogastropoda, Vetigastropoda, Neritimorpha, basal caenogastropods, and basal heterobranchs) or missing (Cocculiniformia).

In the present study, we report 10 new, complete, mitochondrial genomes of pulmonates, with a special focus on lineages that were poorly or not sampled (Figure 2, Table 1): *Salinator rhamphidia *(Amphiboloidea); *Auriculinella bidentata, Myosotella myosotis, Ovatella vulcani*, and *Pedipes pedipes *(Ellobiidae); *Peronia peronii *(Onchidiidae); *Siphonaria gigas *(Siphonariidae); *Succinea putris *(Stylommatophora); *Trimusculus reticulatus *(Trimusculidae); and *Rhopalocaulis grandidieri *(Veronicellidae). Here we also provide a set of 94 new, pulmonate-specific primers spanning the entire mitochondrial genome and specifically designed for the present study. These new primers were combined in multiple pairs, or with 10 mitochondrial primers previously published, to amplify genomes through simultaneous, short reactions. This direct approach was of great help for amplifying mitochondrial genomes in the present study. Pulmonate relationships are evaluated through phylogenetic analyses based on these new genomes as well as 17 genomes published previously. The impact of this new data set on our understanding of the relationships and evolution of pulmonates is discussed.

**Table 1:** List of the species included in the present study

Classification	Species Name	Locality	Voucher	Genbank
Caenogastropoda	*Cymatium parthenopeum*	-	-	EU827200
Caenogastropoda	*Ilyanassa obsoleta*	-	-	DQ238598
Caenogastropoda	*Lophiotoma cerithiformis*	-	-	DQ284754
Basal Heterobranchia	*Pyramidella dolabrata*	-	-	AY345054
Pulmonata, Amphibolidae	*Salinator rhamphidia*	Australia, NSW	CASIZ 180470	JN620539*
Pulmonata, Ellobiidae	*Auriculinella bidentata*	Azores	CASIZ 184730	JN606066*
Pulmonata, Ellobiidae	*Myosotella myosotis*	Azores	CASIZ 184731	JN606067*
Pulmonata, Ellobiidae	*Myosotella myosotis*	-	-	AY345053
Pulmonata, Ellobiidae	*Ovatella vulcani*	Azores	CASIZ 180486	JN615139*
Pulmonata, Ellobiidae	*Pedipes pedipes*	Azores	CASIZ 180476	JN615140*
Pulmonata, Hygrophila	*Radix balthica*	-	-	HQ330989
Pulmonata, Hygrophila	*Biomphalaria glabrata*	-	-	AY380531
Pulmonata, Onchidiidae	*Onchidella borealis*	-	-	DQ991936
Pulmonata, Onchidiidae	*Onchidella celtica*	-	-	AY345048
Pulmonata, Onchidiidae	*Peronia peronii*	Guam	CASIZ 180486	JN619346*
Pulmonata, Siphonariidae	*Siphonaria gigas*	Panama	UF 359645	JN627205*
Pulmonata, Siphonariidae	*Siphonaria pectinata*	-	-	AY345049
Pulmonata, Stylommatophora	*Albinaria coerulea*	-	-	X83390
Pulmonata, Stylommatophora	*Succinea putris*	France	CASIZ 180491	JN627206*
Pulmonata Trimusculidae	*Trimusculus reticulatus*	California	CASIZ 177988	JN632509*
Pulmonata, Veronicellidae	*Rhopalocaulis grandidieri*	Madagascar	NM L7086	JN6193467*
Opisthobranchia, Anaspidea	*Aplysia californica*	-	-	AY569552
Opisthobranchia, Cephalaspidea	*Hydatina physis*	-	-	DQ991932
Opisthobranchia, Cephalaspidea	*Pupa strigosa*	-	-	AB028237
Opisthobranchia, Notaspidea	*Berthellina ilisima*	-	-	DQ991929
Opisthobranchia, Nudibranchia	*Chromodoris magnifica*	-	-	DQ991931
Opisthobranchia, Sacoglossa	*Ascobulla fragilis*	-	-	AY345022

Locality data and museum catalogue numbers of vouchers are indicated for the genomes newly sequenced here. Abbreviations for museum collections holding the vouchers are: California Academy of Sciences, San Francisco, United States of America (CASIZ); Natal Museum, Pietermaritzburg, South Africa (NM); Florida Museum of Natural History, University of Florida, Gainesville, Florida, United States of America (UF). An asterisk (*) next to a Genbank accession number indicates the 10 complete genome sequenced for the present study.

## Results

### Genome structural features

The structural features of each of the 10 mitochondrial genomes sequenced in this study are summarized in Table 2. Each genome consists of 13 protein-coding genes, two rRNA genes, and 22 tRNA genes. The genomes vary in size from 13, 968 bp (*Peronia peronii*) to 16, 708 bp (*Pedipes pedipes*) with most in the range of 14, 000 bp. In all 10 genomes, 13 of the 37 genes are coded on the minus strand: *trnQ, trnL2, atp8, trnN, atp6, trnR, trnE, rrnS, trnM, nad3, trnS2, trnT*, and *cox3*. In *Succinea putris, trnY *and *trnW *are also coded on the minus strand, as well as *trnH *in *Siphonaria gigas*. In the majority of the protein-coding genes (59 of 130), the start codon is TTG. Alternatively, the start codon is either ATG (43), GTG (17), ATT (6), ATA (3), CTG (1), or TTA (1). The stop codon is either TAA (52), TAG (41), T (36), or TA (1), none of which is used solely for a particular protein-coding gene.

**Table 2:** Structural features of the 10 new mitochondrial genomes

	*Auriculinella* *bidentata*	*Myosotella* *myosotis*	*Ovatella* *vulcani*	*Pedipes* *pedipes*	*Peronia* *peronii*	*Salinator* *rhamphidia*	*Succinea* *putris*	*Rhopalocaulis* *grandidieri*	*Trimusculus* *reticulatus*	*Siphonaria* *gigas*
**Total** **size**	14, 135	14, 215	14, 274	16, 708	13, 968	14, 007	14, 092	14, 523	14, 044	14, 518
**%A**	25.8	25.2	25.0	28.6	27.1	26.7	33.8	29.3	26.4	24.3
**%T**	30.9	32.5	29.7	33.7	37.3	35.6	42.9	33.9	34.7	37.2
**%C**	20.4	20.3	21.6	18.4	15.4	16.9	10.8	19.7	18.2	15.1
**%G**	22.6	22.0	23.7	19.3	20.3	20.8	12.1	17.1	20.6	23.4
**%A+T**	56.7	57.7	54.7	62.3	64.4	62.3	76.7	63.2	61.1	61.5
**POR**	44	46	44	? (397)	54	44	47	42	47	72
***rrnL***	1, 037	1, 041	1, 057	1, 138	1, 033	1, 025	1, 020	1, 006	1, 057	1, 109
***rrnS***	704	714	711	786	714	714	755	716	719	755
***cob***	1, 111 (TTG/T)	1, 110 (TTG/TAG)	1, 110 (TTG/TAA)	1, 108 (TTG/T)	1, 108 (TTG/T)	1, 111 (TTG/T)	1, 107 (TTG/TAG)	1, 068 (TTG/TAG)	1, 110 (TTG/TAA)	1, 125 (TTG/TAA)
***cox1***	1, 533 (TTG/TAA)	1, 527 (ATG/TAA)	1, 533 (TTG/TAA)	1, 527 (TTG/TAA)	1, 525 (TTG/T)	1, 527 (TTG/TAA)	1, 548 (TTG/TAG)	1, 527 (TTG/TAA)	1, 527 (TTG/TAG)	1, 530 (GTG/TAG)
***cox2***	687 (TTG/TAG)	669 (GTG/TAA)	666 (TTG/TAG)	681 (GTG/TAA)	666 (TTG/TAA)	664 (GTG/T)	649 (ATG/T)	634 (TTG/T)	666 (TTG/TAA)	672 (GTG/TAA)
***cox3***	778 (ATG/T)	780 (ATG/TAG)	778 (ATG/T)	780 (GTG/TAG)	804 (ATG/T)	781 (ATG/T)	783 (ATG/TAG)	772 (ATG/T)	778 (ATG/T)	810 (ATG/TAA)
***nad1***	906 (GTG/TAA)	906 (TTG/TAG)	906 (TTG/TAA)	903 (ATG/TAG)	906 (TTG/TAA)	958 (TTG/T)	916 (TTG/T)	919 (TTG/T)	906 (TTG/TAA)	903 (TTG/TAA)
***nad2***	945 (ATG/TAG)	948 (ATG/TAG)	942 (CTG/T)	927 (ATG/TAA)	939 (GTG/TAG)	925 (TTG/T)	975 (TTG/TAA)	915 (GTG/TAA)	916 (TTG/T)	942 (ATG/TAA)
***nad3***	354 (TTG/TAA)	349 (TTG/TAA)	354 (TTG/TAA)	372 (ATG/TAA)	327 (ATT/TAA)	349 (TTG/T)	352 (ATG/T)	351 (GTG/TAA)	357 (ATG/TAG)	361 (ATG/T)
***nad4***	1, 311 (TTG/TAG)	1, 305 (TTG/TAA)	1, 308 (TTG/TAG)	1, 302 (ATG/TAG)	1, 318 (TTG/T)	1, 311 (TTG/TAG)	1, 326 (ATG/TAA)	1, 308 (ATG/TAA)	1, 306 (TTG/T)	1, 324 (TTG/T)
***nad4L***	327 (TTG/TAG)	291 (TTG/TAA)	286 (TTG/T)	288 (GTG/TAG)	283 (ATG/T)	279 (TTG/TAG)	275 (ATA/TA)	274 (TTG/T)	286 (ATG/T)	294 (GTG/TAA)
***nad5***	1, 665 (ATA/TAG)	1, 689 (ATA/TAG)	1, 671 (TTG/TAG)	1, 701 (ATG/TAG)	1, 671 (TTG/TAG)	1, 671 (TTG/TAG)	1, 680 (ATG/TAA)	1, 662 (ATT/TAA)	1, 680 (TTG/TAG)	1, 674 (GTG/TAA)
***nad6***	483 (ATT/TAA)	477 (TTG/TAA)	480 (ATT/TAG)	462 (TTG/TAA)	468 (ATT/TAA)	474 (TTG/TAA)	453 (ATG/TAA)	450 (ATG/TAG)	456 (TTG/TAG)	489 (ATT/TAG)
***atp6***	643 (GTG/T)	645 (ATG/TAG)	645 (GTG/TAG)	642 (ATG/TAG)	642 (TTG/TAA)	643 (ATG/T)	657 (ATG/TAA)	618 (ATG/TAA)	643 (ATG/T)	657 (ATG/TAA)
***atp8***	157 (GTG/T)	153 (ATG/TAG)	159 (ATG/TAG)	153 (ATG/TAA)	153 (ATG/TAA)	151 (ATG/T)	123 (TTG/TAA)	151 (GTG/T)	186 (ATG/TAG)	162 (ATG/TAA)

The size of each genome and the POR are in bp. Start and stop codons for protein-coding genes are indicated in parentheses.

All 10 mitochondrial genomes have adjacent overlapping genes, typically 11 to 12 genes; the highest number (15) of overlapping genes is found in *Peronia peronii *and the fewest number (7) is found in *Pedipes pedipes*, which is likely correlated with genome size. The amount of overlap between genes is typically between 1 and 30 bp. Only one pair of overlapping genes is common to all 10 mitochondrial genomes: *trnK, cox1 *(5 to 8 bp). With the exception of *Pedipes pedipes*, the other nine mitochondrial genomes all have *nad6, nad5 *(2 to 18 bp) overlapping and *nad5, nad1 *(14 to 26 bp) overlapping. The largest overlaps in adjacent genes are 39 bp between *nad2, trnK *in *Succinea putris *and 45 bp between *nad4L, cob *in *Auriculinella bidentata*. The number of intergenic spacers range from four in *Peronia peronii *to 20 in *Pedipes pedipes*. The typical size of the intergenic spacers varies from 1 to 80 bp. A large intergenic spacer (651 bp) is found in *Rhopalocaulis grandidieri *between *trnS1, trnS2*, and another one (270 bp) in *Ovatella vulcani *between *trnM, nad3*. Of the 20 intergenic spacers found in *Pedipes pedipes*, five of them are sizeable and AT-rich: 288 bp between *nad6, nad5*, 447 bp between *trnS2, trnT*, 397 bp between *cox3, trnQ*, 700 bp between *trnR, trnS1*, and 317 between *nad4, trnI*. An AT-rich intergenic spacer between *cox3, trnI *is found in all 10 mitochondrial genomes, and this was determined to be the potential origin of replication (POR) which concurs with previous findings [6]. Due to gene rearrangements, the POR for *Pedipes pedipes *may be located between *cox3, trnQ *(397 bp) or between *nad4, trnI *(317 bp). The POR of *Pedipes pedipes *is likely adjacent to the start of *cox3 *for two reasons. First, the percentage of A+T in the 50 bp adjacent to *cox3 *is higher than the 50 bp adjacent to *trnI *(62.0% and 58.8%, respectively). Second, a POR adjacent to *cox3 *was previously found in other gastropod mitochondrial genomes [10].

The 22 tRNAs for each of the 10 mitochondrial genomes were easily located, with the exception of *trnS1 *in *Rhopalocaulis grandidieri*. Unlike the *trnS1 *of the other nine mitochondrial genomes, the anticodon loop sequence is CTGCTAG instead of the typical CTGCTAA and there are two base mispairings in the anticodon loop, which is also uncommon (region sequenced with a 4X coverage). All of the *trnS1 *and *trnS2 *genes lack the DHU stem.

### Molecular phylogeny

**Figure 3:**
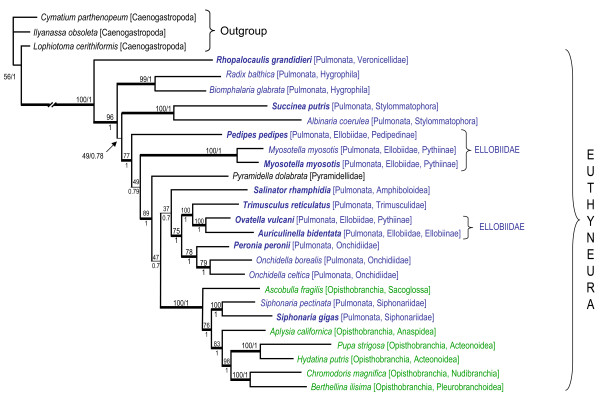
**Phylogenetic relationships within euthyneurans (pulmonates and opisthobranchs)**. Topology obtained from the ML and BI analyses, based on the concatenated amino acid sequences of the 13 protein-coding genes. Opisthobranchs are indicated in green and pulmonates in blue. Node support values are cited using the following format: ''100/1'' means that ML bootstrap value = 100%, and that BI posterior probability = 1.00. Branch lengths depicted here are from the Bayesian analysis (-LnL = -99753.12) performed with MTRev+I+G model using Mr Bayes in Topali. The ML bootstrap values indicated here are from the ML analysis performed with PhyML in Topali (MTRev+I+G, log-likelihood -99834.23). Alternative analyses provided similar topology, branch lengths, and support values.

The trees from the ML and Bayesian analyses share identical topologies and similar branch lengths and node support values (Figure 3). Pulmonates form a paraphyletic group at the base of the Euthyneura; *Pyramidella dolabrata*, traditionally regarded as a basal heterobranch external to Euthyneura, is nested within (paraphyletic) pulmonates. *Siphonaria*, traditionally regarded as a basal pulmonate, is nested within Opisthobranchia which form a derived, monophyletic group at the tip of the euthyneuran tree. The veronicellid slug *Rhopalocaulis grandidieri *is the most basal species of the euthyneurans, with high node support. Freshwater snails *Biomphalaria glabrata *and *Radix balthica *form a clade emerging just after *Rhopalocaulis grandidieri*. Land snails *Succinea putris *and *Albinaria coerulea *form a clade emerging just after the freshwater snails. The ellobiids (*Pedipes pedipes, Myosotella myosotis, Ovatella vulcani*, and *Auriculinella bidentata*) are not monophyletic and are spread throughout the basal portion of the tree. However, the false limpet *Trimusculus reticulatus *(Trimusculidae) is recovered as closely related to two ellobiids (*Ovatella vulcani *and *Auriculinella bidentata*), which is strongly supported. The three species of onchidiids (*Peronia peronii, Onchidella borealis*, and *Onchidella celtica*) form a highly-supported monophyletic group. *Salinator rhamphidia *(Amphiboloidea), traditionally regarded as a basal pulmonate (mainly because of the presence of an operculum), is not basal with respect to all pulmonates. Within the clade Opisthobranchia, *Ascobulla fragilis *is found to be the most basal. AU and SH tests performed indicate that other alternative hypotheses of monophyly (Eupulmonata, Geophila, Ellobiidae, and Systellommatophora) are rejected (Table 3).

**Table 3:** Statistical tests of alternative phylogenetic relationships

Topology *	Log-likelihood	AU	SH
**Unconstrained: **(Rh, ((Ra, Bi), ((Su, Al), (Ped, ((Mm, Mm), (Pyr, ((Sal, ((Tr, (Ov, Au)), (Per, (Ob, Oc)))), (As, ((Sp, Sg), (Ap, ((Pu, Hy)(Ch, Be))))))))))))	-99834.23	0.99	0.99

**Eupulmonata: **(Pyr, (As, ((S.p, S.g), (Ap, ((Pu, Hy)(Ch, Be))))), (Sal, (Tr, ((Ra, Bi), (Rh,, ((Su, Al), (Ped, ((Mm, Mm), ((Ov, Au), (Per, (Ob, Oc)))))))))))	-99872.19	< 0.01	< 0.01

**Geophila: **(Pyr, (As, ((Sp, Sg), (Ap, ((Pu, Hy)(Ch, Be))))), (Sal, (Tr, ((Ra, Bi), ((Ped, ((Mm, Mm), ((Ov, Au), (Rh, ((Su, Al), (Per, (Ob, Oc))))))))))))	-99929.87	< 0.01	0.01

**Ellobiidae: **(Pyr, (As, ((Sp, Sg), (Ap, ((Pu, Hy)(Ch, Be))))), (Sal, (Tr, ((Ra, Bi), ((Ped, ((Mm, Mm), (Ov, Au))), (Rh, ((Su, Al), (Per, (Ob, Oc)))))))))	-99956.03	< 0.01	0.01

**Systellommatophora: **(Pyr, (As, ((Sp, Sg), (Ap, ((Pu, Hy)(Ch, Be))))), (Sal, (Tr, ((Ra, Bi), ((Ped, ((Mm, Mm), (Ov, Au))), (((Per, (Ob, Oc)), Rh), (Su, Al)))))))	-999997.54	< 0.01	< 0.01

*: Rh = *Rhopalocaulis grandidieri*; Ra = *Radix balthica*; Bi = *Biomphalaria glabrata*; Su = *Succinea putris*; Al = *Albinaria coerulea*; Ped = *Pedipes pedipes*; Mm = *Myosotella myosotis*; Pyr = *Pyramidella dolabrata*; Sal = *Salinator rhampidia*; Tr = *Trimusculus reticulatus*; Ov = *Ovatella vulcani*; Au = *Auriculinella bidentata*; Per = *Peronia peronii*; Ob = *Onchidella borealis*; Oc = *Onchidella celtica*; As = *Ascobulla fragilis*; Sg = *Siphonaria gigas*; Sp = *Siphonaria pectinata*; Ap = *Aplysia californica*; Pu = *Pupa strigosa*; Hy = *Hydatina putris*; Ch = *Chromodoris magnifica*; Be = *Berthellina ilisima*.

Some nodes are strongly supported, with Bayesian posterior probabilities of 1 and bootstrap values of 100%, such as the monophyly of opisthobranchs (including *Siphonaria*) and the monophyly of the clade including *Trimusculus *and two ellobiids (*Ovatella *and *Auriculinella*). Some other nodes are still reasonably supported, although less strongly, with Bayesian posterior probabilities of 1 and bootstrap values superior to 75%. However, four of the deeper nodes display no statistical support, with Bayesian posterior probabilities less than 0.8 and bootstrap values less than to 50%. Those nodes, indicated as thin lines in Figure 3, should be regarded as unresolved polytomies, which directly affects the interpretation of the relationships (see Discussion).

### Genome organization and rearrangements

**Figure 4:**
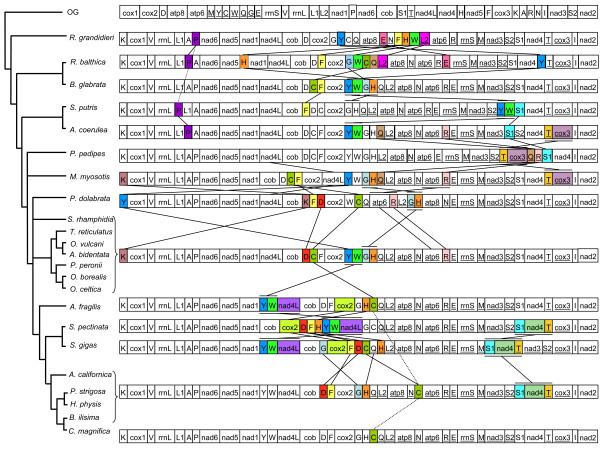
**Hypothesized gene rearrangements of the genomes included in the present study**. The phylogenetic topology is from the present study. Genes encoded by the minus strand are underlined.

*Biomphalaria glabrata, Salinator rhamphidia, Trimusculus reticulatus, Ovatella vulcani, Auriculinella bidentata, Peronia peronii, Onchidella borealis, Onchidella celtica *share an identical mitochondrial genome organization (Figure 4). *Albinaria coerulea *shares the same genome organization except that the position of *trnS1 *and *trnS2 *are switched and *trnS1 *is inverted. The genome of the two freshwater snails *Radix balthica *and *Biomphalaria glabrata *differ in the location of five tRNAs (*P, H, G, C*, and *Y*). The genome of *Rhopalocaulis grandidieri *differs in the location of seven tRNAs (*C, F, G, W, H, L2*, and *E*). The genome of *Succinea putris *differs in the location of three tRNAs (*F, Y*, and *W*), with the latter two genes being coded on the minus strand instead of the plus strand. In *Pedipes pedipes, trnT, cox3 *swapped with *trnS1, nad4*, and *trnQ *and *trnR *moved between these two swapped sets of genes. *Myosotella myosotis *only differs from the most standard mitochondrial genome organization by the rearrangement of *nad4L *between *cox2 *and *trnY*. The location of *trnY *before *cox1 *is a unique and unusual feature of the mitochondrial genome of *Pyramidella dolabrata*. Other attributes, such as the location of *atp6 *prior to *atp8 *and the encoding of *trnG *by the minus strand, are exclusive to *Pyramidella dolabrata*.

The opisthobranch genomes only differ from the common mitochondrial genome arrangement in the position of *trnY, trnW*, and *trnC*. The *trnC *is located between *trnH *and *trnQ *in *Ascobulla fragilis, Chromodoris magnifica*, and *Berthellina ilisima*, and between *trnN *and *atp6 *in *Aplysia californica, Pupa strigosa*, and *Hydatina physis*. The organization of the genome of *Siphonaria gigas *is more similar to the genome of opisthobranchs than pulmonates, which is also true for *Siphonaria pectinata *despite some moderate rearrangements (e.g., *trnY *and *trnW *are adjacent to *nad4L*).

## Discussion

Species diversity of the pulmonate gastropods is largely dominated by the land snails and slugs, or Stylommatophora, which include at least 25, 000 species. However, less than five percent of the pulmonate species diversity is found in the ten other higher taxa, which are anatomically and ecologically very distinct from each other: Amphiboloidea, Ellobiidae, Hygrophila, Onchidiidae, Otinidae, Siphonariidae, Smeagolidae, Trimusculidae, Veronicellidae, and Williamiidae. The present contribution constitutes a significant increase in taxon sampling for complete mitochondrial genomes of pulmonates, especially regarding the non-stylommatophoran taxa (Figure 2, Table 1): complete mitochondrial genomes of amphiboloids, trimusculids, veronicellids, and two ellobiid "subfamilies" (Pedipedinae and Ellobiinae) are presented here for the first time. As of today, complete mitochondrial genomes are unavailable for only three of the pulmonate higher taxa: Otinidae (only one species known), Williamiidae (less than 10 species), and Smeagolidae (less than 10 species).

The topology of the consensus tree produced by our phylogenetic analyses (Figure 3) is very similar to the trees obtained previously based on all protein-coding genes of the mitochondrial genomes [6,7]: pulmonates are paraphyletic, at the base of euthyneurans (opisthobranchs and pulmonates); opisthobranchs (including *Siphonaria*) are monophyletic, at the tip of the tree. *Pyramidella dolabrata*, traditionally regarded as a basal heterobranch (outside euthyneurans), is more closely related to pulmonates. *Biomphalaria glabrata *is found here to be at the base of pulmonates [7] instead of at a more derived position in the tree [6].

The trees based on mitochondrial genomes (Figure 3) show some congruence with the trees based on individual genes -18S, 28S, 16S, COI (Figure 1). In particular, both data sets agree on some nodes that were unsuspected before, such as the close relationship of *Trimusculus reticulatus *and ellobiids. However, both data sets also show some noticeable differences. This might be explained by the fact that some of the deeper nodes are poorly supported by the mitochondrial AA data set presented here (Figure 3). Also, the taxon sampling differs greatly: nearly 80 species of pulmonates are included in a recent analysis using 18S, 16S, and COI sequences [5] while only 20 complete mitochondrial genomes of pulmonates are available, including those generated in this study. Below we discuss the impact of our phylogenetic results on our understanding of the evolution of pulmonate gastropods. In particular, we focus on the new insights and new questions raised by the addition of the 10 new mitochondrial genomes.

In the present results, the most basal lineage of all euthyneurans are the terrestrial, veronicellid slugs, represented here by one species, *Rhopalocaulis grandidieri *(Figure 3). Traditionally, Veronicellidae has been classified in Systellommatophora, along with Onchidiidae and Rathouisiidae [12], although no synapomorphies could be found for systellomatophorans in cladistic analyses [1]. The representation of veronicellids in molecular analyses is recent, but trees based on individual gene sequences support the monophyly of Systellommatophora (Figure 1) [4,5]. A basal position of veronicellids with respect to other pulmonates was proposed based on morphology [13,14]. However, this result was based on problematic interpretations of the anatomy of systellommatophorans, such as the fact that they supposedly lack a pneumostome and a lung. If confirmed, a basal position of veronicellids could lead us to re-interpret their extremely reduced pallial cavity. However, additional mitochondrial genomes of veronicellids will be necessary for testing this new result, because a single genome might introduce a bias (e.g., long-branch attraction) in the analyses.

Freshwater snails (here represented by *Radix balthica *and *Biomphalaria glabrata*) are found to be among the most basal lineages, although their relationships with respect to Stylommatophora (here represented by *Succinea putris *and *Albinaria coerulea*), are unclear. However, the position of Hygrophila is still quite unstable and does vary from one study to another [2,4,5]. For unclear reasons, our attempts to get new complete genomes of freshwater pulmonates failed (very few long PCRs worked), although we started with fresh material from 13 species).

A relatively basal position of Stylommatophora with respect to other pulmonates was obtained in previous analyses based on mitochondrial genomes and individual genes (Figure 1) [5-7]. Many derived features characterize land snails and slugs, especially in the excretory and pulmonary systems, which are physiologically critical for life on land [1,12]. As a result, land snails and slugs have often been regarded as the most derived -and thus most recent - lineage of pulmonates. However, several of those derived features are autapomorphic (e.g., ureter anatomy) or potentially homoplasic (e.g., eyes at the tip of the eye tentacles) [1]. A basal position of land snails and slugs supports Solem's theory according to which stylommatophorans were the first pulmonates to emerge 350 Mya [5,12]. Although stylommatophorans are among the most basal pulmonates and euthyneurans here, their relationships with respect to freshwater snails (Hygrophila) are unclear because of low node support.

The Ellobiidae is a diverse taxon with about 800 species names available in the literature, although only 250 of them are likely to be valid. More importantly, Ellobiidae is phylogenetically diverse because the 24 ellobiid genera are characterized by different combinations of plesiomorphic and derived characters [15,16]. As a result, no exclusive morphological synapomorphy can be found for ellobiids within the broader context of all pulmonates [1]. For many years, molecular analyses could not adequately test the phylogenetic status of ellobiids because of low taxon sampling [2,6]. However, ellobiids were found to be monophyletic in a recent molecular analysis based on a new, comprehensive data set including 25 ellobiid species (Figure 1) [5], suggesting that additional sequenced mitochondrial genomes might be needed to properly address the phylogenetic status of the Ellobiidae, especially considering that two subfamilies (Carychiinae and Melampodinae) are still not represented.

Although the phylogenetic status of Ellobiidae remains problematic, analyses based on concatenated mitochondrial AA sequences and those based on individual genes (18S, 28S, 16S, COI) all agree that the false limpet *Trimusculus reticulatus *is closely related to ellobiids (Figure 1) [2-5]. *Trimusculus reticulatus *also shares the same genome organization as several ellobiid species (Figure 4). This is a new result that had not been suggested using morphology. In fact, there is not any obvious anatomical feature that appears to be shared by *Trimusculus *and ellobiids [5]. Some features are found in ellobiids and *Trimusculus*, but they are also found in many other pulmonates, such as the globineurons and a hypoathroid central nervous system [1,17]. Another interesting result obtained here, as well as in recent analyses based on individual genes, is the close relationship between Onchidiidae and some ellobiids (Figure 1). Onchidiidae and Ellobiidae were suggested to be classified together in the Ellobioidea [18], partly based on characters from the nervous system.

Our present data (Table 3) reject the Geophila hypothesis (Systellommatophora and Stylommatophora) which is also not supported by other recent molecular data (Figure 1). Morphological data supported the monophyly of Geophila, mainly based on two synapomorphies, i.e., the loss of heterostrophy and the presence of eyes at the tip of the ocular tentacles [1]. Both of these features might have evolved independently in Stylommatophora, Onchidiidae, and Veronicellidae. The Eupulmonata hypothesis *sensu *Morton (Geophila and Ellobiidae) also is rejected here [15].

The present analyses confirm two important results from recent molecular studies (Figure 1) [2-6]. First, Amphiboloidea, traditionally regarded as one of the most basal lineages of pulmonates, is not basal, suggesting that their operculum (amphiboloids and glacidorbids are the only pulmonates with an operculum) was acquired secondarily. Second, the pyramidelloids, traditionally regarded as basal (i.e., non-euthyneuran) heterobranchs, are now consistently found to be nested within pulmonates [2-5,19]. However, the pyramidelloids have always been difficult to classify. Some past authors have even regarded them as opisthobranchs [20-23]. The fact that pyramidellids differ so much from other pulmonates may be related to the fact that they live submerged in the seawater (*Williamia *species are the only other pulmonates that live submerged as adults).

Finally, the present data confirm that *Siphonaria *is more closely related to opisthobranchs than to pulmonates, although this relationship is much clearer with mitochondrial genomes than with individual genes (18S, 28S, 16S, and CO1) [2-6,8]. More specifically, *Siphonaria *appears to be most closely related to sacoglossans (here represented as *Ascobulla fragilis*). For most of the 20^th ^century, authors have classified *Siphonaria *within Pulmonata, mainly because they tend to live in the upper intertidal zone, exposed to the air for most of the day, unlike opisthobranchs, which all live submerged even when found in the intertidal zone. As a result, the pallial cavity of *Siphonaria *has been interpreted as a pulmonary cavity and their gills as secondary gills. However, early anatomists had recognized the similarity of the pallial gill of *Siphonaria *with that of cephalaspideans [24,25], suggesting that they could be homologous [1,26]. The pallial gill of *Siphonaria *was even sometimes referred to as a cephalaspidean gill [27]. *Siphonaria *and opisthobranchs share another important feature, i.e., the production of a milky white substance (polypropionate metabolites) when irritated [28], although defensive metabolites are also found in other pulmonates, such as *Onchidium *(Onchidiidae) and *Trimusculus *[29]. Even the gene order of the mitochondrial genome of *Siphonaria gigas *(see the position of *trnY, trnW, nad4L*, and *cob*; Figure 4) supports an affinity with opisthobranchs.

The order of the protein-coding, rRNA, and tRNA genes in mitochondrial genomes is known to be potentially informative for phylogenetics [30]. However, the genomes of pulmonate gastropods display limited informative variation because the organization of protein-coding and ribosomal genes is stable across euthyneurans (Figure 4). Also, Figure 3, which places gene order in a phylogenetic context, suggests that species may share the same gene order because of convergence. Indeed, the freshwater snail *Biomphalaria glabrata *shares the same gene order as other distantly-related pulmonates represented here (*Salinator, Trimusculus, Ovatella, Auriculinella, Onchidella*, and *Peronia*), but differs from the other freshwater snail represented here, *Radix balthica*, for five tRNA genes. So, the gene order found in *Biomphalaria glabrata *and other pulmonates likely is due to convergence, i.e., tRNA genes ended in identical positions through distinct but convergent series of rearrangements. The fact that the genomes of the two *Siphonaria *species differ in the position of two protein-coding genes (*nad4 *and *nad4L*), which has been exceptionally observed in euthyneurans, supports the same idea that the gene order of the mitochondrial genome of a given species should probably not be used to extrapolate on the gene order of a whole lineage. In caenogastropods, Rawlings and collaborators also demonstrated that two closely-related species of *Dendropoma *can differ significantly in genome organization [11]. When additional genomes are available for each major lineage of pulmonate (Veronicellidae, Hygrophila, Onchidiidae, etc.), it might become possible to determine a common gene order to each lineage, but it is not possible at this stage because of our still limited number of complete genomes. There is little doubt, however, that, as additional complete genomes become available in euthyneurans, we will discover an increased variability in euthyneuran gene order.

## Conclusion

The present data constitute a significant increase in taxon sampling for complete mitochondrial genomes of pulmonates (Figure 2). However, despite these efforts, we are still far from a comprehensive understanding of higher relationships of pulmonates. Most of the deep nodes are still uncertain, mainly due to low support values as well as some incongruence between analyses based on different data sets (complete mitochondrial genomes *versus *individual genes -18S, 28S, 16S, CO1), these two issues being obviously related (Figures 1, 3). Additional pulmonate genomes are needed, especially for the taxa for which no genome is currently available (*Williamia, Otina*, and *Smeagol*). Euthyneuran relationships would also greatly benefit from the addition of new genomes of basal heterobranchs (Architectonicoidea, Valvatoidea, Omalogyroidea, Rissoelloidea, Orbitestellidae). Being the most closely related taxa to euthyneurans, basal heterobranchs could help stabilize the topology within euthyneurans.

Euthyneuran phylogenetics and, more broadly, molluscan phylogenetics are still based on limited data, at least compared with vertebrate or arthropod phylogenetic studies which may include more than 40 kb of sequence data. Addressing some of the incongruence between trees based on individual genes (18S, 28S, 16S, CO1) and those based on complete mitochondrial genomes in euthyneuran phylogenetics is a long-term goal that is beyond the scope of the present study. Progress will obviously require increased taxon sampling, but, more importantly, new markers, especially given that, in some ways, using mitochondrial genomes is similar to analyzing one marker with many loci [5]. In the future, when the number and diversity of molecular sequences and taxon samples are increased, merging all data into a single data set may also help provide greater resolution.

Meanwhile, we have to accept that our progress regarding euthyneuran phylogenetics will be slow. Step by step, important discoveries are being made (Figures 1 and 3). Some of the results obtained from recent molecular studies constitute new, major findings [2-5]. In particular, all data sets support close relationships between the false limpet *Trimusculus reticulatus *and ellobiids. Also, pyramidelloids, regarded for many decades as basal (non-euthyneuran) heterobranchs, are nested within pulmonates. Finally, the false limpets *Siphonaria*, traditionally regarded as pulmonates, are closely related to the sacoglossan opisthobranchs; mitochondrial genomes strongly suggest that *Siphonaria *is nested within the monophyletic Opisthobranchia (Figure 3), while individual genes suggest that *Siphonaria *and sacoglossans could be at the base of the monophyletic Pulmonata (Figure 1). All those results reinforce the idea that opisthobranch and pulmonate phylogenetics and evolution should be studied together, as euthyneurans [1,26].

## Methods

### Taxon Sampling

In addition to the ten mitochondrial genomes successfully sequenced for the present study, our analyses also include 17 complete mitochondrial genomes of gastropods obtained from GenBank (Table 1). Of the ten mitochondrial genomes of pulmonates available (Figure 2), three were not selected here: the sequence of *Cepaea nemoralis*, generated more than 15 years ago, is of poor quality; the mitochondrial genomes of *Biomphalaria tenagophila *and *Platevindex mortoni*, although publicly available in Genbank, are still unpublished and should be published shortly by their authors. In addition, seven genomes were selected to represent the major lineages of opisthobranchs (Anaspidea, Cephalaspidea, Notaspidea, Nudibranchia, and Sacoglossa). Three outgroups were selected within Caenogastropoda.

### Species identification, Vouchers, and DNA extraction

Species for which genomes were obtained in the present study were identified by taxonomic experts (some of whom are co-authors of the present article): Rosemary Golding identified the *Salinator*, Suzete Gomes *Rhopalocaulis*, Antonio M. de Frias Martins the ellobiids, Tracy White the *Siphonaria*, and Benoît Dayrat the *Peronia, Succinea*, and *Trimusculus*. Voucher specimens are deposited in museum collections (Table 1). For each species, the complete mitochondrial genome was obtained from a single individual (with the exception of the tiny *Pedipes pedipes *for which three individuals had to be used). In most cases, that individual is part of the lot deposited as voucher. However, the small specimen used for DNA extraction of *Salinator, Auriculinella*, and *Ovatella *had to be destroyed. In these cases, the voucher lot contains other individuals from the same population. DNA was extracted using the phenol-chloroform extraction protocol with cetyltrimethyl-ammonium bromide (CTAB) [31].

### PCR Amplification and Sequencing

Three approaches were combined to successfully obtain complete mitochondrial genomes (Additional file 1): 1) long PCR products (from ~3 to ~10 kb) were amplified and sequenced using shotgun sequencing; 2) short PCR products (less than ~1.5 kb) were amplified using pulmonate-specific primers spanning the entire genome and specifically designed for the present study (pulmonate-specific primers were designed through the alignment of all the sequences of pulmonate mitochondrial genomes available when the present study started); 3) short PCR products were amplified by 'primer-walking, ' i.e., using individual-specific primers designed in previously sequenced regions (by individual-specific primer, we mean that distinct primers were designed for each genome being sequenced). Sequence coverage ranged from 2X (sequences from pulmonate- and individual-specific primers) to greater than 20X (sequences from shotgun sequencing of long PCRs). Only high-quality chromatograms were utilized in areas where sequence coverage was 2X. Additional file 1 summarizes how these three approaches were combined to obtain each genome.

#### Long PCR amplification

Five pairs of universal primers were utilized to generate short PCR products within five mitochondrial genes: *cox1, cox3, cob, rrnS*, and *rrnL *[32]. In order to increase the success of long PCR amplification, individual-specific primers were designed within the short PCR fragments of *cox1, cox3, cob, rrnS*, and *rrnL *obtained with universal primers. Different combinations of individual-specific primers were then used to amplify large regions of the genome (from ~3 to ~10 kb). In some cases, universal primers were combined with individual-specific primers (combinations of two universal primers very rarely yielded successful amplifications). The 25 μl short PCR reactions contained 10.9 μl of water, 2.5 μl of 10X PCR Buffer, 2 μl of 25 mM MgCl_2_, 1 μl of each 10 μM primer, 2 μl of dNTP Mixture, 0.2 μl (1 unit) of TaKaRa Taq (Code No. R001A), 5 μl of 20 ng/μl template DNA, and 0.4 μl of 100X BSA (10 mg/ml Bovine Serum Albumin). In some short PCR reactions, BSA was replaced with 5 μl of 5X Qiagen Q-Solution (water added to these reactions was 6.3 μl). The thermoprofile used for *cox3, cob*, and *rrnS *was five minutes at 94°C; 40 cycles of 40 seconds at 94°C, 1 minute at 46°C, and 1 minute at 72°C; and 10 minutes at 72°C. The thermoprofile used for *cox1 *and *rrnL *was five minutes at 94°C; 30 cycles of 40 seconds at 94°C, 1 minute at 46°C, and 1 minute at 72°C; and 10 minutes at 72°C. The 25 μl long PCR reactions contained 3.8 μl of water, 2.5 μl of 10X LA PCR Buffer II, 0.5 μl of 25 mM MgCl_2_, 5 μl of each 2 μM primer, 3 μl of dNTP Mixture, 0.2 μl (1 unit) of TaKaRa LA Taq (Code No. RR002M), and 5 μl of 20 ng/μl template DNA. The thermocycler profile for the long PCRs consisted of one minute at 98°C, followed by 30 cycles of 98°C for 10 seconds and 68°C for 15 minutes, with a final extension of 10 minutes at 72°C. All long and short PCR products were cleaned with Qiagen QIAquick PCR Purification Kit (Cat. No. 28106) prior to sequencing.

#### Shotgun sequencing of long PCR products

The purified long PCR products were sheared using the HydroShear from GeneMachines. The sheared DNA was then blunt-end repaired and visualized on a 1% agarose gel stained with ethidium bromide. Gel fragments of the correct size (~250bp) were excised and purified from the gel. The repaired DNA fragments were then ligated into the pmcl vector plasmid and transformed into competent *Escherichia coli *cells by electroporation. The cells were then blue/white screened on agar plates and 96 white colonies were picked to form each clone library. A random selection of the library was then amplified by PCR to ensure the DNA insert was present. The libraries were sequenced at the Joint Genome Institute (JGI), Walnut Creek, California, in the context of a Genomics course taught by Dr. Mónica Medina, in collaboration between the University of California at Merced and the JGI.

#### Short (pulmonate-specific) PCR amplification

Only one genome was sequenced in its entirety using only long PCR and shotgun sequencing (*Trimusculus reticulatus*; see Additional file 1). For six other genomes, long PCR and shotgun sequencing yielded partial genomes with gaps of various sizes that needed to be closed. A set of 94 pulmonate-specific primers were designed (Additional file 2) to close those gaps in the six partial mitochondrial genomes obtained through shotgun sequencing. Multiple combinations of pulmonate-specific primers were used to amplify gaps in partial genomes obtained from shotgun sequencing. Those combinations were also used to amplify genomes of species without going through shotgun sequencing (see, Additional file 1). In some cases, depending on the strand used for transcription, a combination of two forward primers (e.g. *Nad4*-879F and *Cox3*-164F) or two reverse primers (e.g. *Nad3*-128R and *Nad4*-642R) had to be used. For some individuals (e.g. *Salinator rhamphidia *and *Rhopalocaulis grandidieri*), the long PCR product was used as the template for amplification instead of the genomic DNA (for increasing chances of successful amplification). For those PCRs targeting gaps of ~900 bp or greater, the long PCR reaction and thermoprofile was used (see above). Short PCR reactions (see above) were used for gaps less than 900 bp with the following thermoprofile: two minutes at 94°C; 5 cycles of 40 seconds at 94°C, 45 seconds at 45°C, and 1 minute at 72°C; 30 cycles of 40 seconds at 94°C, 40 seconds at 55°C, and 1 minute at 72°C; and 3 minutes at 72°C. PCR products were cleaned with either the Qiagen QIAquick PCR Purification Kit or ExoSAP (2 μl of 1u/μl Shrimp Alkaline Phophatase, 0.1 μl of 20u/μl Exonuclease I, and 6 μl of water per 4 μl of PCR product) and sent for sequencing. Some PCR products proved difficult to sequence directly (typically longer PCR products) and were cloned using the Promega pGEM-T Easy Vector System II (Cat. No. A1380) then cleaned with the Promega Wizard Plus SV Minipreps DNA purification System (Cat. No. A1330) prior to sequencing (products were sent out for sequencing).

#### Short (individual-specific) PCR amplification

For the gaps that remained in the mitochondrial genomes after using the pulmonate-specific primers and shotgun sequencing, individual-specific primers were designed and the gaps were primer-walked until completion of the genome (Additional file 1). In some instances, a combination of a pulmonate-specific and an individual-specific primer was used in an attempt to close gaps. PCR conditions for the individual-specific primers were identical to those used for the pulmonate-specific primers.

### Genome assembly and annotation

The shotgun sequence chromatograms produced by the JGI were read, bases were called, and a value was assigned to the quality of the called bases by the Phred program [33]. The individual sequences were assembled into contigs using Phrap http://www.phrap.org. The contigs created by Phrap were analyzed and assembled to form longer, more complete contigs using Consed [34]. Contig sequences from the shotgun sequencing were saved in MacVector with Assembler 9.5.2 http://www.macvector.com. All other sequences (i.e., all sequences not obtained through sequencing at the JGI; see Additional file 1) were assembled in MacVector.

Open reading frames (ORFs) of the assembled contigs were analyzed by MacVector and the tentative identity of each protein-coding gene was determined based on the map of the mitochondrial genome of *Aplysia dactylomela*. Each gene was added to its corresponding Se-Al v2.0a11 http://evolve.zoo.ox.ac.uk file containing the alignment of multiple homologous gastropods sequences and the gene was demarcated on the genome based on the results of the alignment. The limits of both the protein-coding and rRNA genes were adjusted manually based on location of adjacent genes. All tRNA genes were located by hand based on the anticodon and the fairly conserved anticodon stem and loop sequence.

### Phylogenetic analyses

The 13 protein-coding gene sequences were first translated into amino acid sequences and then individually aligned using the default parameters of ClustalW (1.6) in MEGA version 4.1 (Beta) [35]. Each alignment was cropped to remove variation on either end and then sequences were concatenated. The concatenated protein-coding genes alignment (3, 458 amino acids) was adjusted manually and a minimal number of sites were then removed (gaps created by insertions in the sequences of the caenogastropod outgroups). Three caenogastropod species were used as outgroups: *Cymatium parthenopeum, Ilyanassa obsoleta*, and *Lophiotoma cerithiformis *(Table 1).

Because genes of animal mitochondrial genomes are fast evolving [36-38], phylogenetic analyses based on amino acid sequences are commonly preferred to analyses based on nucleotide sequences [6,7,39]. Also, it has been shown that individual genes provide less topological resolution than concatenated genes [10].

The amino acid substitution model was determined to be MTRev+I+G using the Akaike Information Criterion (AIC) in Topali version 2.5 [40]. The same model was obtained whether each gene was treated independently as a distinct partition, or all genes were concatenated in a single data set. Maximum likelihood analysis of the amino acid was run using the MTRev+I+G model with PhyML in Topali, with bootstrap support values based on 1000 replicates. An alternative ML analysis was also run in RAxML [41] using MTzoa [42], a model specifically designed for lophotrochozoan mitochondrial data sets. Bayesian Metropolis-coupled Markov chain Monte Carlo (MCMC) analysis was performed on the amino acid sequences using the MTRev+I+G model with MrBayes in the Topali interface (two parallel analyses, 100 million generations, sampled every 100 generations, 25% burn-in). An alternative Bayesian analysis was performed using the MTzoa model. The Tracer v1.4 [43] graphical tool was used to visualize convergence of the chains from Bayesian analyses. In addition, posterior samples from different (five) runs were compared, which is arguably the best approach to detect potential problems of chain convergence [44].

Alternative phylogenetic relationships were tested with the Approximately Unbiased (AU) and Shimodaira-Hasegawa (SH) tests, using CONSEL [45] using default settings. Alternative relationships were based on traditional, morphology-based results as well as recent molecular results [1,5,26].

## Competing interests

The authors declare that they have no competing interests.

## Authors' contributions

TW participated in the molecular work and genome annotation, performed phylogenetic analyses, and wrote a first draft of the manuscript. MC and SB participated in the molecular work and RT in the genome annotation. RG and AFM provided fresh samples and commented on the manuscript. RT and SB participated in the research while undergraduates at the University of California at Merced. BD designed and developed the entire project. All authors read and approved the final manuscript.

## Supplementary Material

Additional file 1 **Approaches used to obtain the 10 new mitochondrial genomes in the present study**. Three approaches were combined to get each of the 10 mitochondrial genomes of the present study: 1) shotgun sequencing; 2) sequencing of short and long PCR fragments obtained with pulmonate-specific primers (designed for the present study; see Additional file 2); 3) sequencing of short and long PCR fragments obtained with individual-specific primers (designed for the present study). The asterisk (*) indicates individuals in which the long PCR product was used as the template for the majority of PCR reactions using pulmonate-specific and individual-specific primers. Click here for file

Additional file 2 **Pulmonate-specific primers designed for the present study**. Those pulmonate-specific primers were designed: 1) by building alignments for the sequences of each individual gene (except for tRNAs) from the complete, pulmonate, mitochondrial genomes available prior to the present study (Figure 2, Table 1), and 2) by locating conserved regions. All primers were specifically designed for the present study, with the exception of ten of them: in *cox1*, F14 and R698 [59]; in *rrnL*, F437 and R972 [60]; in *cob*, F384 and R827 [61]; in *rrnS*, F302 and R695 [60]; in *cox3*, F174 and R713 [61]. Click here for file
