# Supplementary material for: Ten new complete mitochondrial genomes of pulmonates (Mollusca: Gastropoda) and their impact on phylogenetic relationships
Source: BMC Evol Biol. 2011 Oct 10;11:295. doi: 10.1186/1471-2148-11-295 (PMC3198971; doi:10.1186/1471-2148-11-295)
Supplement: Additional file 1 — Approaches used to obtain the 10 new mitochondrial genomes in the present study. Three approaches were combined to get each of the 10 mitochondrial genomes of the present study: 1) shotgun sequencing; 2) sequencing of short and long PCR fragments obtained with pulmonate-specific primers (designed for the present study; see Additional file 2); 3) sequencing of short and long PCR fragments obtained with individual-specific primers (designed for the present study). The asterisk (*) indicates individuals in which the long PCR product was used as the template for the majority of PCR reactions using pulmonate-specific and individual-specific primers. [file 1471-2148-11-295-S1.DOC]

**Additional file 1**

**Approaches used to obtain the 10 new mitochondrial genomes in the present study.** Three approaches were combined to get each of the 10 mitochondrial genomes of the present study: 1) shotgun sequencing; 2) sequencing of short and long PCR fragments obtained with pulmonate-specific primers (designed for the present study; see Additional file 2); 3) sequencing of short and long PCR fragments obtained with individual-specific primers (designed for the present study). The asterisk (*) indicates individuals in which the long PCR product was used as the template for the majority of PCR reactions using pulmonate-specific and individual-specific primers.

| **Species** | **JGI Shotgun Sequencing** | **Pulmonate-Specific Primers** | **Individual-Specific Primers** |
| --- | --- | --- | --- |
| *Auriculinella bidentata* | *cox3* to *nad1* (6,735bp), *cob* to *cox3* (6,012bp) |  | *nad1* to *cob* (1,803bp), *nad4* to *nad2* (1,274bp) |
| *Myosotella myosotis* | *trnM* to *trnN* (13,249bp) |  | *trnN* to *trnM* (1,996bp) |
| *Ovatella vulcani* | *nad3* to *cox1* (4,372bp), *cox1* to *trnY* (7,041bp) | *cox1* to *rrnL* (1,618bp) | *cox2* to *nad3* (2,691bp) |
| *Pedipes pedipes* | *cox1* to *cob* (7,474bp), *rrnS* to *nad2* (6,102bp) |  | *nad2* to *cox1* (1,343bp), *cob* to *rrnS* (~3,000bp) |
| *Peronia peronii* | *cox1* to *cox2* (6,705bp) | Combination of pulmonate-specific and individual- specific primers (~7,300bp) from *cox2* to *cox1* | |
| *Rhopalocaulis grandidieri** |  | *nad2* to *rrnL* (~2,850), 16S to *nad6* (~900bp), *nad1* to *nad4L* (~860bp), *cob* (~600bp), *cob* to *cox2* (~750bp), *cox2* to *atp6* (~600bp), *atp6* to *rrnS* (~500bp), 12S to *nad3* (~980bp), *nad4* to *nad2* (~2500bp) | Remaining gaps |
| *Salinator rhamphidia** |  | Combination of pulmonate-specific and individual-specific primers for the entire genome | |
| *Siphonaria gigas* |  | *cox1* to *nad6* (~2,700bp), middle of *cob* (~500bp), *atp6* to *nad4* (~2,300bp), *cox3* to *nad2* (~650bp), *nad2* to *trnK* (~650bp) | Remaining gaps |
| *Succinea putris* | *cox3* to *trnH* (4,585bp), *cob* to *cox2* (1,197bp) | Combination of pulmonate-specific and individual-specific primers to fill remaining gaps (~8,300bp) | |
| *Trimusculus reticulatus* | Complete sequence |  |  |
