# Supplementary material for: Ten new complete mitochondrial genomes of pulmonates (Mollusca: Gastropoda) and their impact on phylogenetic relationships
Source: BMC Evol Biol. 2011 Oct 10;11:295. doi: 10.1186/1471-2148-11-295 (PMC3198971; doi:10.1186/1471-2148-11-295)
Supplement: Additional file 2 — Pulmonate-specific primers designed for the present study. Those pulmonate-specific primers were designed: 1) by building alignments for the sequences of each individual gene (except for tRNAs) from the complete, pulmonate, mitochondrial genomes available prior to the present study (Figure 2, Table 1), and 2) by locating conserved regions. All primers were specifically designed for the present study, with the exception of ten of them: in cox1, F14 and R698 [59]; in rrnL, F437 and R972 [60]; in cob, F384 and R827 [61]; in rrnS, F302 and R695 [60]; in cox3, F174 and R713 [61]. [file 1471-2148-11-295-S2.DOC]

**Additional File 2**

**Pulmonate-specific primers designed for the present study.** Those pulmonate-specific primers were designed: 1) by building alignments for the sequences of each individual gene (except for tRNAs) from the complete, pulmonate, mitochondrial genomes available prior to the present study (Figure 2, Table 1), and 2) by locating conserved regions. All primers were specifically designed for the present study, with the exception of ten of them: in *cox1*, F14 and R698 [59]; in *rrnL*, F437 and R972 [60]; in *cob*, F384 and R827 [61]; in *rrnS*, F302 and R695 [60]; in *cox3*, F174 and R713 [61]. Primer sequences are in the 5’ to 3’ direction. The naming system for primers is based on their position in the alignment of each particular gene (after short loose ends were cropped). For example, in *cox1*, the F14 primer refers to a *cox1* forward primer whose 5’ end is at the 14^th^ position in the aligned pulmonate sequences of *cox1*; in the same gene, the R3 primer refers to a reverse primer whose 3’ end is at the 3^rd^ position in the aligned pulmonate sequences of *cox1*. In most cases, the same conserved region was used to design both forward and reverse primers, but not always (to avoid issues such as self priming). Because truly conserved regions are scarce in the mitochondrial genome, most primers are degenerate. For amplification, appropriate combinations must be used, depending on the direction of the transcription of each gene. Each gene is always coded on the same strand across pulmonates, at least from the known mitochondrial genome of pulmonates, except for a few tRNA genes that may be coded on the plus or minus strand depending on the species. As for ribosomal and protein-encoding genes: *rrnL*, *cob*, *cox1*, *cox2*, *nad1*, *nad2*, *nad4*, *nad4L*, *nad5*, and *nad6* are on the plus strand; *rrnS*, *atp6*, *atp8*, *cox3*, and *nad3* are on the minus strand (underlined in the present table). As a result, for amplification, a forward primer and a reverse primer must be combined if the two primers are selected from only one gene (e.g., *cox1*-F253 and *cox1*-R839) or from two genes transcribed in the same direction (e.g., *nad4*-879F and *nad2*-324R); but two forward primers (e.g. *nad4*-879F and *cox3*-164F) or two reverse primers (e.g. *nad3*-128R and *nad4*-642R) must be combined if selected from genes transcribed in opposite directions. In order to estimate the potential length of the PCR products, one should refer to the (quite conserved) pulmonate mitochondrial gene order (Figure 4).

| **Gene** | **Primer** | **Sequence** | **Gene** | **Primer** | **Sequence** |
| --- | --- | --- | --- | --- | --- |
| ***cox1*** | R3 | CCAATRTCTTTRTGRTTHGTNGARWADTCAHCGC | ***cox2*** | F55 | GARATRHTNTNHTNTTYCAYGAYCA |
|  | F14 | WYTCNACDAAYCAYAAAGAYATTGG |  | R55 | TGRTCRTGRAANADNANADYATYTC |
|  | R167 | AYAAANGCRTGDGCWGTMAC |  | F301 | AYHGGNCAYCARTGRTAYTG |
|  | F253 | GGNGCNCCWGAYATRAGHTTYCC |  | R307 | TCRTANSWYCARTAYCAYTGRTG |
|  | R253 | GGRAADCTYATRTCWGGNGCNCC |  | F407 | TNYTWGARGTDGAYAAYCG |
|  | F466 | TTRGGDGCNATTAATTTTATYAC |  | F514 | AARRTRGAYKCDGTNCCNCG |
|  | R466 | GTRATAAAATTAATNGCHCCYAA |  | R514 | CGNGGNACHGMRTCYATTTT |
|  | F698 | TGRTTYTTYGGDCAYCCNGARGTHTAYAT |  | F580 | TAYGGNCARTGYTCNGARAT |
|  | R698 | ATRTADACYTCNGGRTGHCCRAARAAYCA |  | R580 | ATYTCNGARCAYTGNCCRTA |
|  | R839 | GTAAAYATRTGHGCYCANACAATAAAWCC | ***atp6*** | F453 | CGDCCNYTDACHTTRRCNGTDCG |
|  | F844 | GGWTTTATTGTNTGRGCDCAYATRTTTAC |  | R453 | CGHACNGYYAADGTHARNGGHCG |
|  | R1052 | TCYAAHGAWGARTTWGAHARNACAATWCC |  | F483 | GCHAAYATWAGNGCNCAYRTT |
|  | F1055 | GGWATTGTNYTDTCWAAYTCWTCDTTRGA |  | R483 | AAYRTGNGCNCTWATRTTDGC |
|  | F1281 | TAGGDYTDKCWGGHATRCCNCGNCG | ***rrnS*** | F143 | GGTGCCAGCADYCGCGGYCAWACC |
|  | R1281 | CGNCGNGGYATDCCWGMHARHCCTA |  | R143 | TWTGRCCGCGRHTGCTGGCACC |
| ***rrnL*** | F437 | CRNCTGTTTANCAAAAACATAGYY |  | F302 | AAACTRGGATTAGAKACCCYAYTAT |
|  | R437 | RRCTATGTTTTTGNTAAACAG |  | R302 | ATARTRGGGTMTCTAATCCYAGTTT |
|  | F515 | GGCCGCAGTACMYTGACTGTGCDAAGGTAGC |  | F695 | GTGYACAAATCGCCCGTCAYYCY |
|  | R515 | CACAGTCARKGTACTGCGGCC |  | R695 | RGRRTGACGGGCGATTTGTRCAC |
|  | F972 | CCTACATGATCTGAGTTCAGACCGGCGYAAGCCAGGTCAG |  | F735 | AAGTCGTAACAHAGTARRRGTA |
|  | R972 | CCGGTCTGAACTCAGATCATGT |  | R735 | TACYYYTACTDTGTTACGACTT |
| ***nad6*** | R197 | AHADWACHARNADNCCHCCAA | ***nad3*** | F1281 | TTYGARTGYGGNTTYGANCCBHT |
|  | F208 | TTGGDGGNHTNYTDGTWHTDTTTMTTTAT |  | R1281 | ADVGGNTCRAANCCRCAYTCRAA |
| ***nad5*** | F371 | TWGGNTGRGAYGGNYTHGG | ***nad4*** | F401 | TGRGGNTAYCARCCNGARCG |
|  | R371 | CCDARNCCRTCYCANCCTA |  | R401 | CGYTCNGGYTGRTANCCYCA |
|  | R394 | ANNCCDARNCCRTCYCA |  | F642 | CCNAARGCNCAYGTNGARGC |
|  | F639 | TGRYDCCNGCNGCHATRGC |  | R642 | GCYTCNACRTGNGCYTTNGG |
|  | R663 | ARDGCNCTNACNGGNGTNGG |  | F879 | GCHTAYTCNRTDRSNCAYAT |
|  | R940 | ADNGYTTRAAHADNGCRTG |  | R879 | ATRTGNSYHAYNGARTADGC |
| ***nad1*** | R55 | TCNWWHARNGTDWARAADGCNAC |  | F1097 | TNAAYATRGCWGCNCCNCC |
|  | F76b | GARCGNAAGRTNYTAGG |  | R1097 | GGNGGNGCWGCYATRTT |
|  | R76 | CYHARNAYYTTNCGYTC | ***cox3*** | F25 | GTNGARTWYAGHCCHTGRCCNHTWYT |
|  | F419 | GCBCARACWATYTCDTAYGA |  | R25 | ARWADNGGYCADGGDCTRWAYTC |
|  | R419 | TCRTAHGARATWGTYTGVGC |  | F164 | TGRTGRYGDGAYRTHRTHCGWGA |
| ***cob*** | F288 | GGHCGNGGNNTRTAYTAYCA |  | R164 | TCWCGDAYDAYRTCHCRYCAYCA |
|  | R288 | TGRTARTAYANNCCNCGDCC |  | F596 | ACNGGNTTYCAYGGNNYNCAYGT |
|  | F384 | GGNTAYGTNYTDCCDTGRGGNCARAT |  | R596 | ACRTGNRNNCCRTGRAANCCNGT |
|  | R399 | GCNCCYCARWADGAYATYTGNCCCYCA |  | F713 | TGRTAYTGRCAYTTYGTHGAYGT |
|  | F809 | CARCCNGARTGRTAYTTYYTNTTYGCNTAYGC |  | R713 | ACRTCDACRAARTGYCARTAYCA |
|  | R809 | GCRTANGCRAANARRAARTAYCAYTCNGG | ***nad2*** | F324 | TTYCCNKGBCAYTTYTGRGT |
|  | R806 | TANGCRAANARRAARTAYCAYTCNGGYTG |  | R324 | ACYCARAARTGVCMNGGRAA |
|  | R827 | GGAATNGMHCGNARRAYDGCRTANGCRAA |  | F718 | GGNHTNCCHCCHTTYHTHMTVTT |
|  |  |  |  | R718 | AABAKDADRAADGGDGG |
